# Inhibiting Inducible Nitric Oxide Synthase with 1400W Reduces Soman (GD)-Induced Ferroptosis in Long-Term Epilepsy-Associated Neuropathology: Structural and Functional Magnetic Resonance Imaging Correlations with Neurobehavior and Brain Pathology[Fn fn4]

**DOI:** 10.1124/jpet.123.001929

**Published:** 2024-02

**Authors:** Marson Putra, Suraj S. Vasanthi, Nikhil S. Rao, Christina Meyer, Madison Van Otterloo, Lal Thangi, Daniel R. Thedens, Sridhar S. Kannurpatti, Thimmasettappa Thippeswamy

**Affiliations:** Department of Biomedical Sciences, College of Veterinary Medicine, Iowa State University, Ames, Iowa (M.P., S.S.V., N.S.R., C.M., M.V.O., L.T., T.T.); Department of Radiology, Carver College of Medicine, The University of Iowa, Iowa City, Iowa (D.R.T.); and Department of Radiology, Rutgers Biomedical and Health Sciences, New Jersey Medical School, Newark, New Jersey (S.S.K.)

## Abstract

Organophosphate (OP) nerve agent (OPNA) intoxication leads to long-term brain dysfunctions. The ineffectiveness of current treatments for OPNA intoxication prompts a quest for the investigation of the mechanism and an alternative effective therapeutic approach. Our previous studies on 1400W, a highly selective inducible nitric oxide synthase (iNOS) inhibitor, showed improvement in epilepsy and seizure-induced brain pathology in rat models of kainate and OP intoxication. In this study, magnetic resonance imaging (MRI) modalities, behavioral outcomes, and biomarkers were comprehensively investigated for brain abnormalities following soman (GD) intoxication in a rat model. T1 and T2 MRI robustly identified pathologic microchanges in brain structures associated with GD toxicity, and 1400W suppressed those aberrant alterations. Moreover, functional network reduction was evident in the cortex, hippocampus, and thalamus after GD exposure, and 1400W rescued the losses except in the thalamus. Behavioral tests showed protection by 1400W against GD-induced memory dysfunction, which also correlated with the extent of brain pathology observed in structural and functional MRIs. GD exposure upregulated iron-laden glial cells and ferritin levels in the brain and serum, 1400W decreased ferritin levels in the epileptic foci in the brain but not in the serum. The levels of brain ferritin also correlated with MRI parameters. Further, 1400W mitigated the overproduction of nitroxidative markers after GD exposure. Overall, this study provides direct evidence for the relationships of structural and functional MRI modalities with behavioral and molecular abnormalities following GD exposure and the neuroprotective effect of an iNOS inhibitor, 1400W.

**SIGNIFICANT STATEMENT:**

Our studies demonstrate the MRI microchanges in the brain following GD toxicity, which strongly correlate with neurobehavioral performances and iron homeostasis. The inhibition of iNOS with 1400W mitigates GD-induced cognitive decline, iron dysregulation, and aberrant brain MRI findings.:

## Graphical abstract

**Figure:**
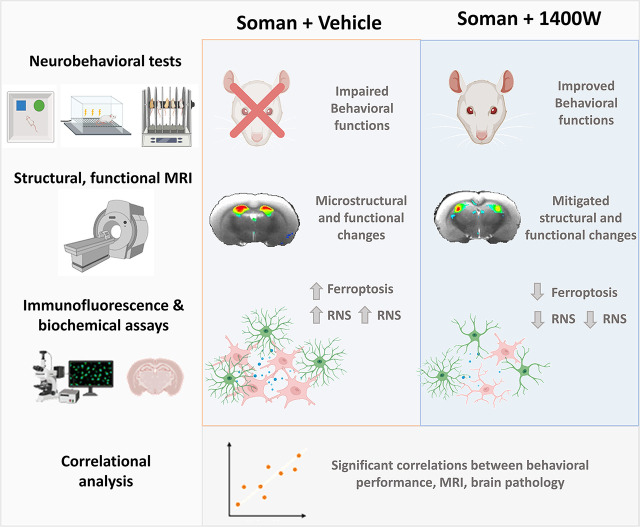

## Introduction

Acute exposure to organophosphate nerve agent (OPNA) induces rapidly progressing status epilepticus (SE) due to cholinergic crisis, which could lead to the development of epilepsy, seizures-associated brain pathology, and neuropsychiatric dysfunction (McDonough and Shih, 1997; Miyaki et al., 2005; Vale et al., 2018; Henretig et al., 2019). Current treatments are inadequate to prevent SE-induced neuropathology by OPNA, necessitating a quest for alternative treatments (Collombet, 2011; Jett, 2016). Administering medical countermeasures within 20 minutes of OPNA exposure is recommended for maximum protection from irreversible brain damage, which is often unrealistic. Thus, developing more effective and practical therapy to complement the current medical countermeasures (atropine, oxime, and benzodiazepines) is deemed significant (Jett, 2016; Jett and Spriggs, 2020). The literature supports the role of nitroxidative stress, neuroinflammation, and neuronal death following OPNAs toxicity (Pazdernik et al., 2001; Guignet et al., 2020; Putra et al., 2020a,c). Overproduction of reactive nitrogen species (RNS) markers, such as neuronal nitric oxide synthase and inducible NOS (iNOS), are shown to mediate seizures-induced extensive brain injuries and functional deficits in epilepsy models (Beamer et al., 2012; Puttachary et al., 2016b), including an organophosphate toxicity model (Putra et al., 2020c). The increase of iNOS levels frequently coincides with excessive reactive oxygen species (ROS) production during epileptogenesis following brain injury, which is evident in the OPNA-induced epilepsy models (Pearson and Patel, 2016; Liang et al., 2019; Putra et al., 2020a). Therefore, nitroxidative-targeted therapy may offer desired neuroprotection against long-term brain pathology in OPNA-induced seizure models. However, the alterations of brain microstructures and network function in epileptic condition following OP exposure and how targeting nitroxidative stress mitigates brain pathologies have not yet been fully evaluated, especially in the soman (GD; a nerve agent) model of epilepsy. Of note, GD refers to a two-character identifier (military designation) for a nerve agent assigned by the North Atlantic Treaty Organization. Structural magnetic resonance imaging (MRI) and functional magnetic resonance imaging (fMRI) in conjunction with behavioral and molecular biomarkers may provide valuable insights into the mechanisms of neuroprotection in the subjects exposed to GD.

MRI is an imperative clinical tool for studying structural and functional brain changes caused by seizures in epilepsy (Kuzniecky et al., 1997; Fu et al., 2021). OPNA-induced seizures result in early pathologic changes in T2 intensity and neuronal damage, with microstructural changes observed in multiple brain regions (Hobson et al., 2018). Although structural MRI changes have been well characterized in OPNA toxicity (Bhagat et al., 2001, 2005; Gullapalli et al., 2010; Reddy et al., 2020), the use of fMRI to assess functional network connectivity and disease modification has not been investigated. We recently reported the differential findings in structural and functional MRI of brains between male and female rats in the GD-induced epilepsy model (Gage et al., 2023). The current study aims to examine the potential disease-modifying effects of an iNOS inhibitor, 1400W, against GD intoxication. We evaluated the changes in multiple brain MRI parameters, cognitive and motor behaviors, and molecular biomarkers of neurodegeneration, including ferritin levels in key brain regions. Iron-mediated brain damage, ferroptosis, is facilitated by reactive microglia and astrocytes following insults in many neurodegenerative diseases, including epilepsy (Tang et al., 2021; Zimmer et al., 2021; Ryan et al., 2023). Although the interaction between iNOS and ferroptosis has been reported to play a major role in promoting inflammation (Tang et al., 2021), whether inhibiting iNOS with 1400W would mitigate ferroptosis and protect the brain in GD-exposed animals is unknown. This study aims to investigate the neuroprotective effects of an iNOS inhibitor, 1400W, against GD intoxication using MRI metrics, memory and motor behaviors, molecular biomarkers, and the relationships between these parameters.

## Materials and Methods

### Animal Source and Care

Adult male and female Sprague-Dawley rats (7 to 8 weeks old; 250–300 g; Charles River Laboratories, Wilmington, MA) were used in the study. Male and female rats were housed in the same room but caged individually in 12-hour light/dark cycles at 22°C with free access to food and water. GD exposure was performed at MRI Global, Kansas City; brain MRI was acquired at the University of Iowa, and the remaining experiments were carried out at Iowa State University. All animal care procedures before and after GD challenge were conducted per the guidelines of National Institutes of Health (NIH) Guide for the Care and Use of Laboratory Animals and approved by the Institutional Animal Care and Use Committee (IACUC) at Iowa State University (IACUC protocol #20-090 and #21-118) and MRI Global (IACUC protocol #23-22). All of the experiments done in this study also comply with the Animal Research Reporting of In Vivo Experiments (ARRIVE) guidelines (Kilkenny et al., 2010).

### Animals Used in This Study

The subset of animals (*N* = 52) used in this correlation study for MRI, behavioral, and ferritin assays were from a large cohort of mixed-sex rats (55 males, 52 females) used in behavioral studies that were reported in our recent publication (Vasanthi et al., 2023). Out of the 52 mixed-sex cohort, 40 animals were used for MRI [eight controls, 16 GD+vehicle (VEH), 16 GD+1400W]. One control, two GD+VEH, and one GD+1400W animals were excluded from MRI analysis due to artifacts developed during scanning. The other 12 animals that were not used for MRI served as controls for histology, western blot, and ELISA.

### GD-Induced SE and Seizure Scoring

**Fig. 1.**
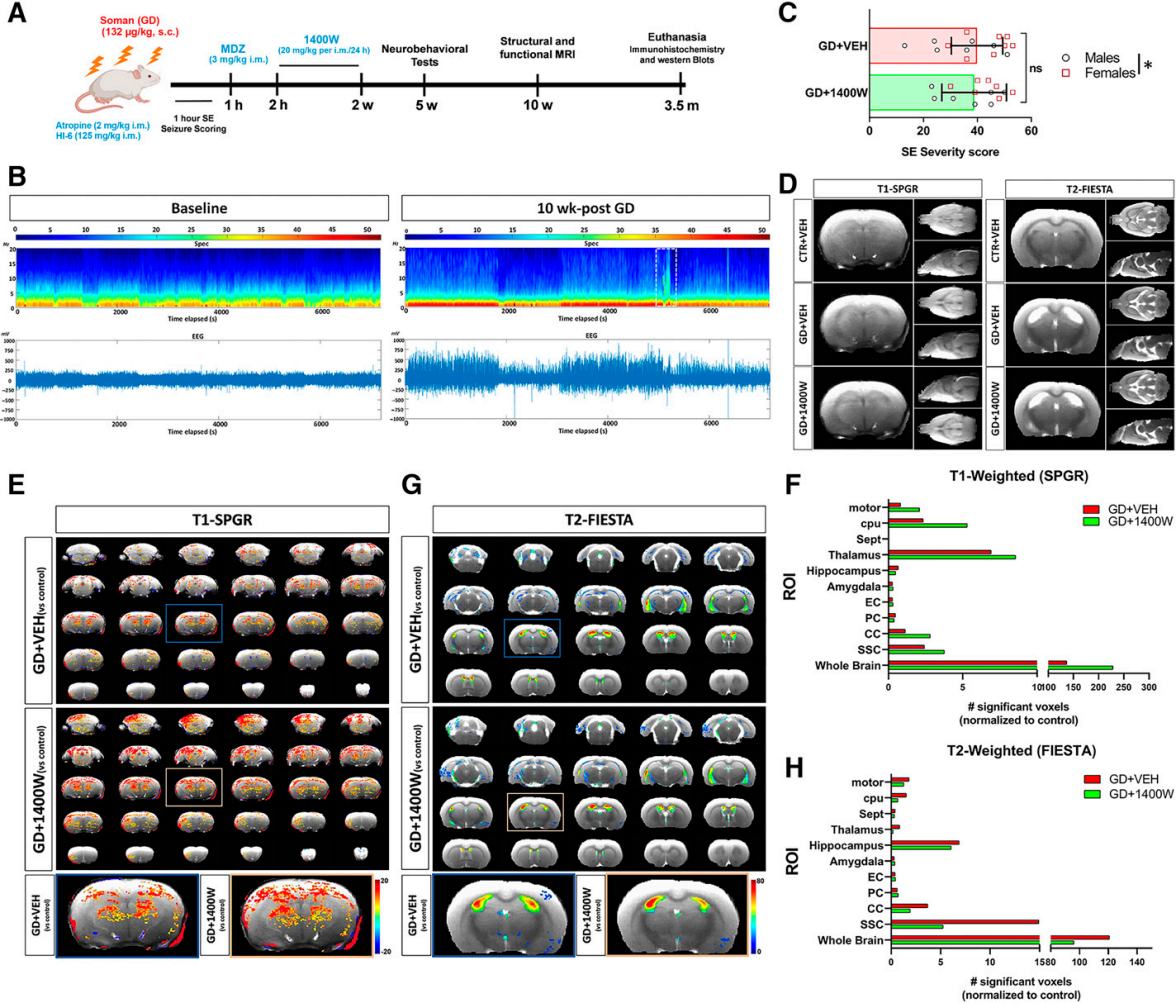
1400W suppresses pathologic changes in structural MRI T1-SPGR and T2-FIESTA. (A) Experimental design. (B) Representative EEG traces and spectrograms comparing the baseline versus 10 weeks post-GD (conducted in a separate study). A spontaneous recurrent seizure episode is shown in a dashed-white box. (C) SE severity comparison between animals (mixed sex) that were later assigned to either the vehicle or 1400W group. There was a significant difference between females (open box) and males (open circle) in behavioral seizure severity within each group. (D) Representative group average T1-SPGR and T2-FIESTA images of control, GD+VEH, and GD+1400W. (E and G) Representative images showing statistically significant T1-SPGR (E) or T2-FIESTA (G) intensity differences between GD+VEH versus Control and GD+1400W versus Control. (F and H) Graphs comparing the number of significant voxels between GD+VEH versus Control and GD+1400W versus Control in multiple brain regions for T1-SPGR (F) or T2-FIESTA (H). Color overlay depicts intensity change magnitudes across statistically significant voxels (*P* < 0.05). A family-wise error control of a minimum cluster of 30 contiguous voxels to correct for multiple comparisons. [*n* = 7 (CTR+VEH), 14 (GD+VEH), 15 (GD+1400W)] . Bar graphs display all data points and are expressed as mean ± S.D. CC, corpus callosum; cpu, caudate putamen; CTR, control; EC, entorhinal cortex; FIESTA, fast imaging employing steady-state acquisition; motor, motor cortex; NS, nonsignificant; PC, perirhinal cortex; sept, septum; SPGR, spoiled gradient recalled; SSC, somatosensory cortex.

Rats were exposed to either vehicle (PBS) or soman (132 µg/kg, s.c., 1.2× LD_50_), followed by treatment with oxime HI-6 dimethanesulfonate (125 mg/kg, i.m.) and atropine sulfate (2 mg/kg, i.m.) within 1 minute to control the peripheral cholinergic effects and mortality. The development of SE was observed and scored for seizure severity for 1 hour. The anticonvulsant midazolam (3 mg/kg, i.m.) was administered 1 hour post GD exposure to control acute behavioral seizures and mortality. The five-point staging scale was used for seizure scoring (i.e., SE severity) as described previously (Putra et al., 2020c): stage 1: excessive salivation, lacrimation, urination, defecation, mastication, and chewing; stage 2: tremors, wet-dog shakes, head nodding, neck jerks, kyphosis, and opisthotonus; stage 3: forelimb clonus, Straub tail, rearing, and rigid extension of forelimbs; stage 4: rearing, forelimb clonus, and loss of righting reflex; and stage 5: abducted limbs clonus/repeated rearing and generalized seizures. Stages 1 and 2 were nonconvulsive seizures, whereas stages 3–5 were considered convulsive seizures. Based on seizure scores during SE, the rats were equally distributed between treatment groups (vehicle or 1400W) to account for matching initial SE severity prior to treatment assignment (Fig. 1C). 1400W (99.6% pure, Tocris Bioscience) was diluted in sterile distilled water at a concentration of 10 mg/mL. Either vehicle (equal volume) or 1400W (20 mg/kg) was given twice daily for the first 3 days, followed by a single dose per day for the next 11 days (a total of 17 doses for 14 days). Following soman exposure, the rats were subjected to neurobehavioral studies, MRI acquisition, immunohistochemistry, and biochemical assays. The experimental design is illustrated in Fig. 1A.

### Neurobehavioral Testing

Animals were subjected to behavioral tests starting at 5 weeks post GD exposure. All behavior procedures were conducted in a noise-free environment by an experimenter blinded to treatment groups. The equipment was thoroughly cleaned with 70% ethanol to remove urine or feces from the previous animal and dried before introducing a new animal. Animals were given rest for 2 to 3 days between each test. The battery of behavioral tests was conducted, starting with the least aversive/stressful test to the most aversive/stressful tests in the following order.

#### Novel Object Recognition.

Novel object recognition (NOR) was used to test hippocampal-dependent spatial learning and recognition memory using a modified protocol (Schartz et al., 2018). The test involves a 3-day procedure: habituation, training, and testing/probing. The rats were habituated in the experimental apparatus for 10 minutes in the absence of objects. The next day, during the training, the animals were placed in the apparatus in the presence of two identical objects (e.g., blocks) and allowed to freely explore for 5 minutes. During the testing period (24 hours post-training), one of the familiar objects was replaced with a novel object, and the rats were placed again in the apparatus for a 5-minute exploration. Training and testing trials were performed blind to treatments, video recorded, and tracked using ANY-maze software (Stoelting Co.). The amount of time spent on object exploration was quantified automatically by the software. The relative object exploration times were expressed as a discrimination index (DI) [DI = (t_novel_ – t_familiar_)/(t_novel_ + t_familiar_)] and compared between groups and sexes.

#### Rotarod.

Rats were tested for motor learning and coordination using an accelerating rotarod apparatus (AccuRotor 4-channel, Omnitech Electronics Inc.) as described previously (Putra et al., 2020a). Briefly, latency-to-fall for each rat was measured on the accelerating rod (5–60 rpm) during each of the three trials (180 seconds) with 5-minute intertrial intervals on training and testing days. Time spent on the rod (latency-to-fall) on the testing day was averaged and compared between groups.

#### Contextual Fear Conditioning.

The rats were tested for fear memory by assessing their ability to recall an association between a conditioned stimulus (a tone) and an aversive stimulus (an electric foot shock). The test had a training (day 1) and a probing (day 2) phase. During training, the rats were individually placed in a soundproof conditioning chamber (Med Associate, Fairfax, VT) for 2 minutes. A 20-second tone (70–80 dB) was then applied, which coterminated with a foot shock (0.75 mA) during the last 2 seconds of the tone. The tone-shock pairings were allowed to occur four times at 80-second intervals. On the second day (24 hours post-training), the rats were placed in the chamber for 2 minutes, followed by four tones (20 seconds each with 80-second intervals) without shock in the same manner as the training day. Time spent freezing (suppression of body movement) in the chamber was automatically quantified with a video-tracking camera and software (Stoelting Co.) and expressed as a percentage of freezing time (% FT). The freezing time (%) increases were measured as [% FT increase = average four FTs (%) post-tone − the FT (%) before the tone].

### Structural and Functional MRI

A week after the behavioral testing, the rats were subjected to brain MRI scanning to investigate the impact of GD exposure on structural and functional changes and mitigation by 1400W. All MRI acquisitions were performed under general anesthesia with isoflurane at 3.0% induction and maintained at 1.0%–1.5% throughout the procedure.

#### Structural MRI.

Brain MRI was acquired using a GE Discovery MR901 7 Tesla horizontal bore scanner. Animals’ heads were secured with ear bars to limit motion and positioned within a volume-transmit radiofrequency (RF) coil of a 7-cm diameter with a four-channel surface coil receiver. T2 steady-state contrast images were obtained using a fast imaging employing steady-state acquisition (FIESTA) sequence for rapid scanning and coverage of the whole brain. MRI parameters used were Repetition Time/Time to Echo (TR/TE) = 4.3 ms/1.9 ms, 108 coronal slices of thickness 0.3 mm, and in-plane resolution of 0.13 mm × 0.13 mm covering a 25-mm × 25-mm field of view. Subsequently, a spoiled gradient recalled echo (SPGR) pulse sequence with TR/TE = 13 ms/2 ms and flip angle 20 degrees acquired 56 slices of 0.6-mm thickness with 0.13-mm × 0.16-mm resolution over a 25-mm × 25-mm field of view to generate T1 weighted images.

#### Functional MRI.

After completion of the structural T1 and T2 sequences, approximately 10 minutes of fMRI measurements were obtained using a T2* weighted gradient recalled echo (GRE)-segmented echo planar imaging (EPI) sequence with blood oxygen level–dependent (BOLD) weighting. The imaging parameters were TR/TE = 1500 ms/15 ms, four segments per image, slice thickness of 0.9 mm, and a field of view 32 mm × 32 mm over a 96 × 96 matrix utilizing 27 slices to cover the whole brain from the olfactory lobe to the cerebellum. A total of 80 image volumes were obtained with a repetition time of 6 seconds per image volume.

#### Image Processing.

All images were obtained in the DICOM format and were reconstructed in the Neuroimaging Informatics Technology Initiative (by the NIH) program for preprocessing and registration using the BioImage suite (Duncan et al., 2004). The sigma rat MRI template (https://www.nitrc.org/projects/sigma_template/) was used as the standard spatial template to which a single control rat brain MRI was nonlinearly registered (50 iterations, normalized mutual information, otherwise default) using the BioImage suite. The resulting study baseline template was used to nonlinearly register all other subject MRIs.

Functional MRI data from all animals were linearly coregistered to the fMRI of the control study subject template and subsequently motion corrected using the fifth image of the respective animal using the Analysis of Functional NeuroImages (Cox, 1996). Resting-state BOLD voxel-level time series data were linearly detrended to remove temporal signal drifts and band-pass filtered between 0.005–0.1 Hz before further analysis. Individual resting-state functional connectivity (RSFC) maps were obtained by seed-based crosscorrelation analysis where the BOLD time series (from a 3 × 3 = 9 voxel volume) was averaged to obtain the mean seed voxel time series and subsequently crosscorrelated across all voxels in the brain. This process was repeated across both hemispheres, yielding two individual RSFC maps per subject. Cortical, hippocampal, and thalamic seed regions were used to obtain the respective RSFC networks across each animal. The average RSFC map in each animal and for each seed region network was determined after converting the correlation coefficients in the individual RSFC maps to z-value using a Fisher z-transform and reconverted to correlation coefficients through an inverse Fisher z-transformation postaveraging as implemented in our previous study (Kannurpatti et al., 2015).

### Tissue Processing and Immunofluorescence Labeling

The animals were euthanized with pentobarbital (100 mg/kg, i.p.) at the end of the study, and the brains were collected as described previously (Putra et al., 2020a). Briefly, transcardiac perfusion with PBS followed by 4% paraformaldehyde in PBS was performed to fix the brain for histology. The brains were postfixed in 4% paraformaldehyde overnight at 4°C, followed by cryoprotection in 25% sucrose for 72 hours at 4°C. The tissues were then gelatin embedded and snap frozen in liquid nitrogen–cooled isopentane. The tissue blocks were either cryosectioned or stored at −80°C. Sixteen-micron coronal sections were cut using a cryostat (NX70, ThermoFisher) and collected on gelatin-coated slides. Our sampling method covered the entire hippocampal and parahippocampal regions from the rostral to the caudal as described previously (Puttachary et al., 2016a). The sections were then permeabilized with 0.2% Triton X-100 and blocked with 10% normal donkey serum. Sections were incubated with primary antibodies of interest for 24 hours at 4°C (Supplemental Table 2). After washing with PBS, the brain sections were incubated with either direct dye-conjugated or biotin-avidin–based secondary antibodies for 1 hour at room temperature (see Supplemental Table 2). Sections were PBS washed and coverslipped using Vectashield (Vector laboratories, CA) water-based mounting medium.

### Microscopy and Cell Quantification

Immunostained sections were visualized using a Leica DMi8 inverted fluorescence microscope (Wetzlar, Germany) and processed using the LASx (version 4.1.2.) software as described previously (Gage et al., 2022). Briefly, the photomicrographs were taken with 20x/0.8 and 40x/1.3 air-objective lenses for analysis and illustration purposes, respectively. Images containing 10 z-stacks at 1.5 μm were obtained and compressed into a maximum contrast projection with the “EBImage” package on R studio Ver 1.11.463 (Bioconductor, MA) software before analysis. The staining intensity expressed as fluorescence intensity value (arbitrary unit) was measured using ImageJ 2.0.0-rc-49/1.51d (NIH). Consistent exposure times were maintained across all images to minimize group variation.

#### Colocalization Analysis.

Image stacks (10 z-stacks, 1.5-μm step size) were imported into ImageJ, and the percent area overlap between two markers [e.g., IBA1 versus ferritin heavy chain 1 (FTH-1) or GFAP versus FTH-1] was quantified by Pearson's correlation coefficient using the JACoP plugin in ImageJ (Dunn et al., 2011).

#### Microglia Morphology Quantification.

A modified version of quantification was developed from the published studies (Young and Morrison, 2018; Putra et al., 2020b). Image stacks (10 z-stacks, 1.5-μm step size) were maximum contrast projected to create a single image with enhanced visualization of microglia processes. Enhanced images were then loaded onto ImageJ software and despeckled to eliminate background noise. The cell body area was determined after converting the pixel into a micrometer scale. The images were binarized and skeletonized with a skeleton analysis plugin (http://imagejdocu.tudor.lu/) to generate data on the number of branches and total length of branches. A minimum of 20 randomly selected microglia in the piriform cortex were analyzed per animal in each group.

## Immunoblotting

The immunoblotting procedure was carried out as previously described (Putra et al., 2020a). Briefly, hippocampal tissues were lysed in a cocktail of radioimmunoprecipitation assay (RIPA) buffer and protease and phosphatase inhibitors (ThermoScientific) at 4°C. Lysates were normalized, resolved on 8% or 10% SDS-PAGE gels, and transferred to a nitrocellulose membrane (Bio-rad). The membrane was then blocked for 1 hour at 25°C with a blocking buffer for fluorescent western blotting (Rockland Immunochemicals). The membrane was incubated with primary antibodies for 24 hours at room temperature. After washing with PBS containing 0.1% Tween 20, the membranes were incubated with infrared dye secondary antibodies, either 680 or 800 nm (Supplemental Table 2), and visualized with LI-COR Odyssey XF imaging system (Li-Cor). Empiria Studio Version 2.2 was used to acquire the images. Quantitative densitometric measurement was performed using ImageJ 2.0.0-rc-49/1.51d (NIH). Complete blot images are available in Supplemental Fig. 3.

## ELISA

Ferritin levels in the sera were determined using ELISA kits (Novus Biologicals, Centennial, CO). The manufacturer’s protocol was followed with minor modifications. Assays were performed in duplicates. Briefly, serum was diluted (1:2 dilution) and added to the wells and incubated for 1.5 hours at room temperature. After washing, the plate was incubated with a primary antibody for ferritin for 1 hour and, after washing, incubated with horseradish peroxidase conjugate for 30 minutes. After adding the stop solution, the samples were estimated for the protein abundance at an optical density of 450 nm using SpectraMax M2 Gemini Molecular Device Microplate reader (Molecular Device, PA). The optical density values were expressed as ferritin concentration in ng/ml.

## Rigor and Statistics

In all experiments, animals were randomized and coded prior to experimentation. Seizure scores during SE were used to equally distribute the rats between treatment groups (vehicle or 1400W) to account for matching initial SE severity prior to treatment assignment (Fig. 1C). All experimenters were blinded to treatment groups. We extracted the behavioral raw data from the large cohort study (Vasanthi et al., 2023) from the same animals that were used for brain MRI acquisition for correlation purposes in this study. Statistical analyses and graphical representations were done in Prism 8.0 (GraphPad Software) and R-Studio version 1.1.463. Normality was evaluated with the Shapiro-Wilk test. Two-way ANOVA was performed to evaluate the interaction between sex and treatment (Vorland, 2021). For multiple group comparisons with no sex interaction, normal data were analyzed using ANOVA, whereas non-normal data were evaluated with the Kruskal-Wallis with appropriate post hoc tests. A mixed-effect model with post hoc was used to analyze treatment effects across brain regions. A two-group comparison was performed using an unpaired *t* test. In cases where sex had an effect, data were analyzed separately for each sex. Correlations were calculated using Spearman correlation coefficients, and slope comparison was performed with ANCOVA. Differences between groups with *P* < 0.05 were considered statistically significant. For voxel-wise MRI comparisons, significant differences between groups were determined with a threshold of *P* < 0.05, corrected for multiple comparisons using a family-wise error control cluster threshold of 20 contiguous voxels. Details of all statistical tests applied in this study are summarized in Supplemental Table 1.

## Results

### 1400W Reduced Brain Pathologic T1 Intensity Decreases and T2 Intensity Increases in Structural MRI.

Abnormal brain structural changes associated with epilepsy have been extensively reported in animal models (Boux et al., 2021) and human patients (Kuzniecky et al., 1997) using T1- and T2-weighted MRI analyses. In our long-term electroencephalogram (EEG) study in the rat GD model (Gage et al., 2022), spontaneous recurrent seizures were evident in most animals by 10 weeks postexposure (Fig. 1A), implying the development of epileptic phenotype. All rats used in the MRI study had handling-induced seizures. The MRI acquisition was performed 1 week after completing the neurobehavioral tests, i.e., 10 weeks post-GD (Fig. 1B). There was no significant difference in SE severity after exposure to GD between animals that were later assigned to vehicle or 1400W. However, there was a significant difference in SE severity between male and female animals (Fig. 1C) used in behavioral and MRI studies.

Structural T1-weighted MRI was acquired and averaged for each group to visually assess intensity changes across the whole brain (Fig. 1D; Supplemental Fig. 1A). T1 intensity changes were initially analyzed for each animal examining the regions of interest (ROIs) between groups, but no significant changes were observed (Supplemental Fig. 1, A–C). Statistical parametric mapping analysis was then performed to track voxel-level changes across groups. Sparse T1 intensity decreases were found across the hippocampus after GD exposure, but significant T1 intensity increases were associated with GD-induced epilepsy in different regions of the brain (Fig. 1, E and F). T1 intensity increases were prominently found in the somatosensory cortex, corpus callosum, hippocampus, thalamus, caudate putamen, and motor cortex. The results suggest fewer T1 intensity decreases in GD-exposed animals receiving 1400W compared with those receiving vehicle, indicating a rescue by 1400W. However, prominent T1 intensity increases due to GD exposure were further enhanced by 1400W following GD intoxication.

T2-weighted MRI was analyzed by examining each animal's average T2 intensity values between groups across all ROIs, but no significant differences were found between groups except in a few brain regions (Fig. 1D; Supplemental Fig. 1D). A linear-mixed effects analysis also showed no difference in T2 intensity changes between groups as a whole. Further voxel-level analysis using statistical parametric mapping revealed significant T2 intensity increases in corresponding brain regions related to GD intoxication. GD animals treated with 1400W showed lesser T2 increases compared with those with vehicle in several brain regions (Fig. 1, G and H). Overall, the structural T1 and T2 MRI changes in 1400W-treated groups indicate that by 10 weeks, GD exposure induces lesser T1 decreases and T2 increases across multiple brain regions.

### 1400W Protected against GD-Induced Loss of Cortical, but Not Thalamic, Functional Connectivity Networks Revealed by fMRI.

**Fig. 2.**
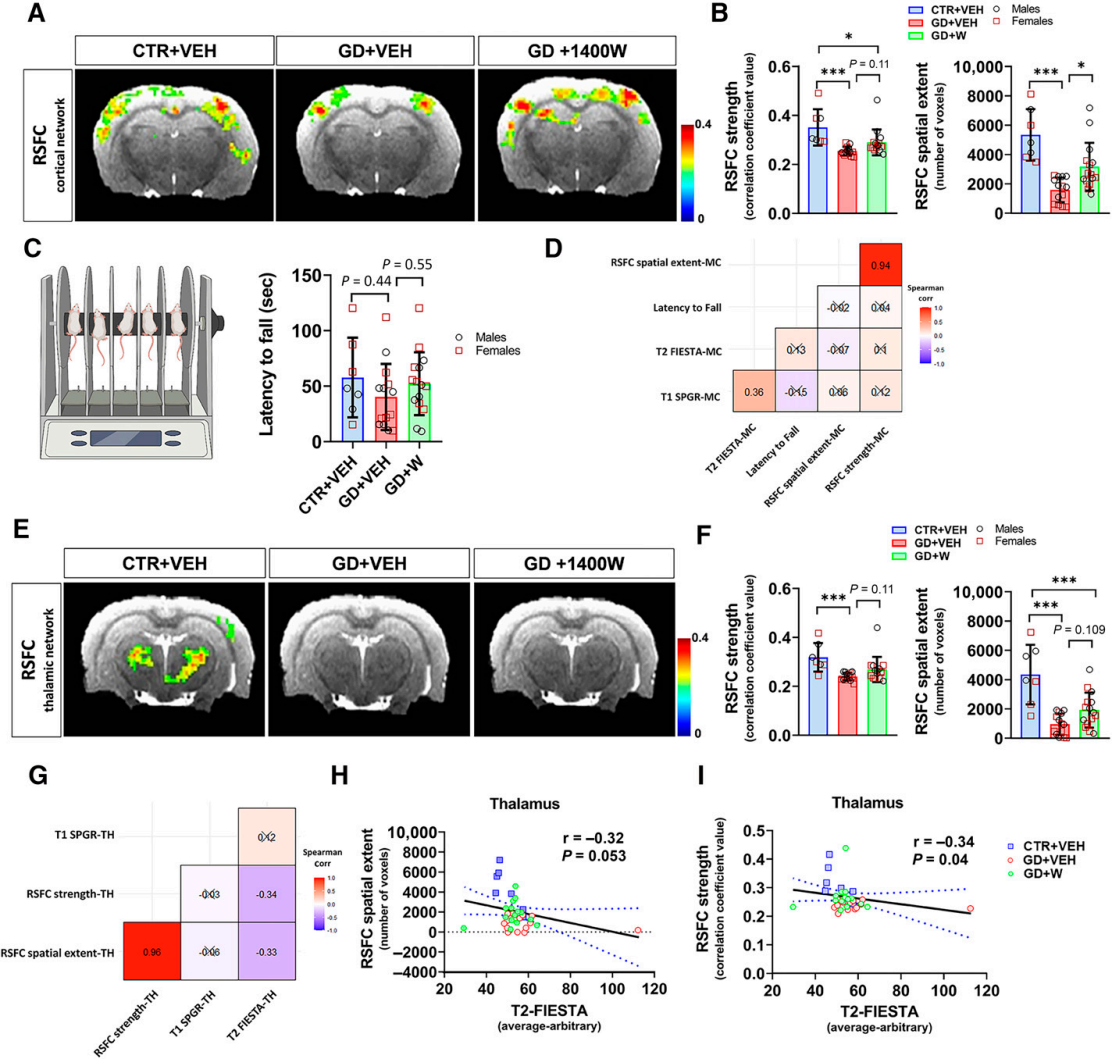
1400W prevents the loss of cortical network but not the thalamic network connectivity following GD intoxication. (A) A representative activation heatmap showing RSFC-cortical network in control, GD+VEH, and GD+1400W. (B) Quantification of cortical RSFC for both strength and spatial extent. (C) Rotarod and quantification of latency-to-fall. (D) Spearman correlation matrix showing no significant correlation between cortical RSFC, T1-SPGR, and T2-FIESTA in motor cortex and latency-to-fall in rotarod. (E) Representative activation heatmaps showing RSFC across the thalamic network. (F) Quantification of thalamic RSFC for both strength and spatial extent. (G) Spearman correlation matrix showing no significant correlation between thalamic (TH) RSFC, T1-SPGR, and T2-FIESTA intensities. (H and I) Spearman correlation analyses between thalamic RSFC spatial extent (H), strength (I), and T2-FIESTA. The blue dotted line in the correlational plots represents a 95% confidence interval for two means. Black crosses (X) in the correlation matrix (D and G) correspond to nonsignificant correlations (r value shown in each box). *n* = 7 (CTR+VEH), 14 (GD+VEH), 15 (GD+W); ∗*P* < 0.05; ∗∗∗*P* < 0.001. Bar graphs display all data points and are expressed as mean ± S.D. No sex differences observed in all graphs. CTR, control; FIESTA, fast imaging employing steady-state acquisition; RSFC, resting-state functional connectivity; SPGR, spoiled gradient recalled; W, 1400W.

**Fig. 3.**
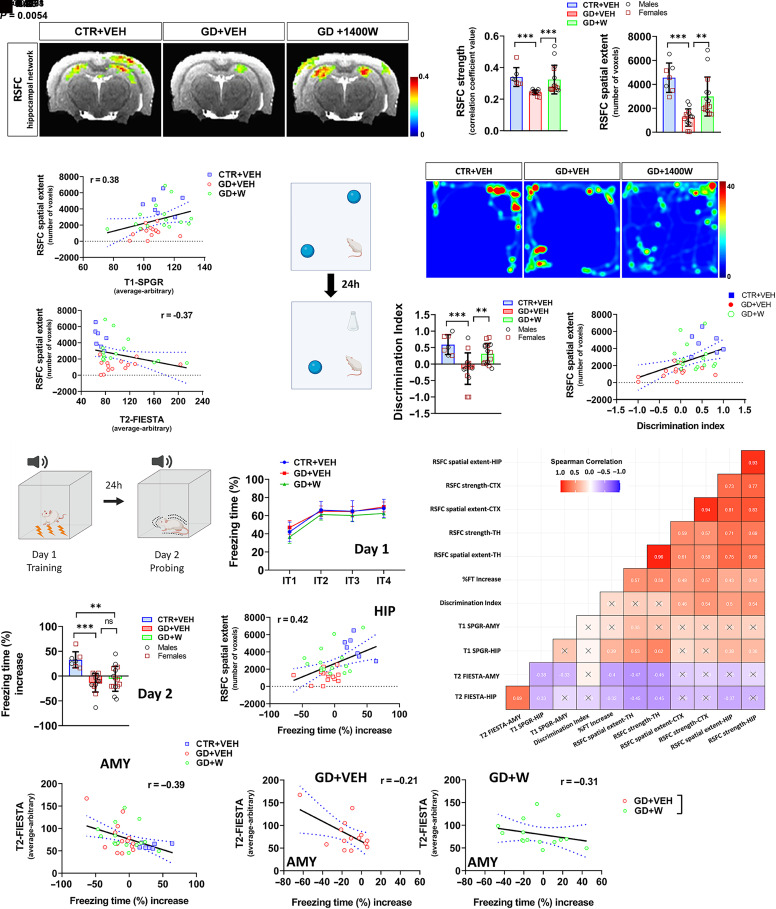
1400W rescues GD-induced hippocampal (HIP) network loss and memory impairment. (A) RSFC-hippocampal network; activation heatmaps depict voxels (*t* test, family-wise error control of minimum cluster of 20 contiguous voxels) for each group. (B) Quantification of RSFC for strength and spatial extent. (C and D) Spearman correlation showing a positive relationship between RSFC spatial extent and hippocampal T1-SPGR (C) and a negative relationship with T2-FIESTA values (D). (E) Experimental design of NOR. (F) Representative heatmaps of the movement track of the animals on the test day. (G) Quantification of DI between groups showing memory impairment by GD and rescue by 1400W. (H) Spearman correlation showing a positive relationship between RSFC spatial extent and DI. (I) Schematic illustrating contextual fear conditioning. (J) On day 1 acquisition, the learning curve for each trial of the tone-shock pair showing no difference between groups. (K) Contextual-cue fear conditioning measuring the difference in the freezing time (%) between before and after the tone (average of four intertrials for each tone) between groups. (L) Spearman correlations showing a positive relationship between freezing time and hippocampal RSFC spatial extent. (M) Spearman correlation of freezing time (%) increase and the amygdala (AMY) T2-FIESTA intensity. (N) Slope comparison for the AMY T2-FIESTA versus freezing time (%) increase in GD+VEH and GD+1400W groups. (O) Heatmap summary of Spearman correlation matrices between the structural, functional MRI parameters, and behavioral outcomes. Black crosses (X) correspond to nonsignificant correlations (r value shown in each box). The blue dots in the correlational plots represent a 95% confidence interval for two means. *n* = 7 (CTR+VEH), 14 (GD+VEH), 15 (GD+W); ∗*P* < 0.05; ∗∗*P* < 0.01; ∗∗∗*P* < 0.001. Bar graphs display all data points and are expressed as mean ± S.D. No sex differences observed in all graphs. CTR, control; CTX, cortex; FIESTA, fast imaging employing steady-state acquisition; RSFC, resting-state fnctional connectivity; SPGR, spoiled gradient recalled; TH, thalamus; W, 1400W.

The abnormal fMRI changes in epileptic brains, particularly in the cortex and thalamus, are associated with neurobehavioral impairment (Woodward and Cascio, 2015; Pressl et al., 2019). Resting-state fMRI was used to measure RSFC in the cortex, thalamus, and hippocampus in GD-induced epilepsy (Figs. 2, A and E and 3A). Both RSFC strength and spatial extent were significantly reduced in GD+VEH, but GD+1400W had a significantly higher cortical RSFC spatial extent, not strength, than GD+VEH (Fig. 2B). There was no significant difference in rotarod performance between control, GD+VEH, and GD+1400W groups, but GD+VEH animals had a trend of lower latency than control animals (Fig. 2C). There was no significant correlation between rotarod performance and T1 and T2 intensity in the motor cortex, and fMRI for cortical connectivity (Fig. 2D).

Similarly, examining the RSFC thalamic network revealed a significant reduction in RSFC strength and spatial extent in the GD+VEH group compared with controls. However, treatment with 1400W did not significantly affect the GD-induced reduction in thalamic RSFC (Fig. 2, E and F). Correlation analysis showed that only T2 intensity values for the thalamus were significantly correlated with thalamic RSFC strength and partly with spatial extent (Fig. 2, G–I). These results suggest partial interdependency of both structural and functional MRI parameters in thalamic regions. Collectively, our data demonstrate that blocking iNOS with 1400W rescues cortical connectivity loss following GD intoxication despite showing no effects on the thalamus.

### 1400W Rescued GD-Induced Loss of Hippocampal Functional Network and Memory Functions.

In the hippocampus, we also observed significantly reduced RSFC strength and spatial extent of GD+VEH compared with control animals. Treatment with 1400W restored this reduction in hippocampal RSFC strength and spatial extent after GD toxicity (Fig. 3, A and B). Correlational analysis showed that the hippocampal RSFC spatial extent was negatively associated with hippocampal T1 decreases and positively associated with T2 increases (Fig. 3, C and D). Our findings suggest that blocking iNOS rescues GD-induced loss of functional hippocampal network, which is strongly associated with structural MRI microchanges.

Exposure to nerve agents induces long-term memory impairment correlated with hippocampal volume loss (Philippens et al., 1992; Reddy et al., 2020). We used the NOR assay to test the effects of GD exposure on memory. The results showed significant deficits in NOR performance of animals treated with GD relative to control animals as reflected in the low DI. 1400W mitigated the impaired memory performance (Fig. 3, E–G). Interestingly, correlation analysis indicated a positive relationship between DI with hippocampal and cortical functional networks (Fig. 3, H and O). Other structural MRI measures had little to no relationship with memory recognition in NOR (Fig. 3O).

To test for associative memory impairment in GD toxicity, we used a fear-conditioning test (Fig. 3I). GD-treated animals receiving either vehicle or 1400W showed significantly impaired memory compared with control animals as demonstrated by lower freezing time (%) increase (Fig. 3, J and K). The correlational analysis demonstrated a significantly positive relationship between RSFC spatial extent for hippocampal connectivity with the freezing time (Fig. 3L), suggesting a primary contribution of the hippocampus. Moreover, strong positive relationships were also observed between thalamic and cortical functional connectivity with freezing time increase (Fig. 3O), indicating broader involvement of multiple brain regions in fear conditioning.

Interestingly, impaired memory in fear conditioning had an inverse relationship with T2 intensity in the amygdala (Fig. 3M). When extrapolating the treatment slopes, we found that the slope for the GD +1400W group was statistically different from the GD+VEH group (Fig. 3N), indicating that protection by iNOS inhibition significantly affects the magnitude and rate of impaired memory in fear conditioning when considering the T2 intensity changes. A correlation matrix showed that the hippocampal network’s RSFC had the most correlation with other MRI parameters in multiple regions of the brain as well as with behavioral outcomes (Fig. 3O), suggesting an interdependence of MRI changes reflected in behavioral measures and partly affected by iNOS inhibition with 1400W.

### 1400W Suppressed GD-Induced Chronic Iron Deposition in the Brain.

**Fig. 4.**
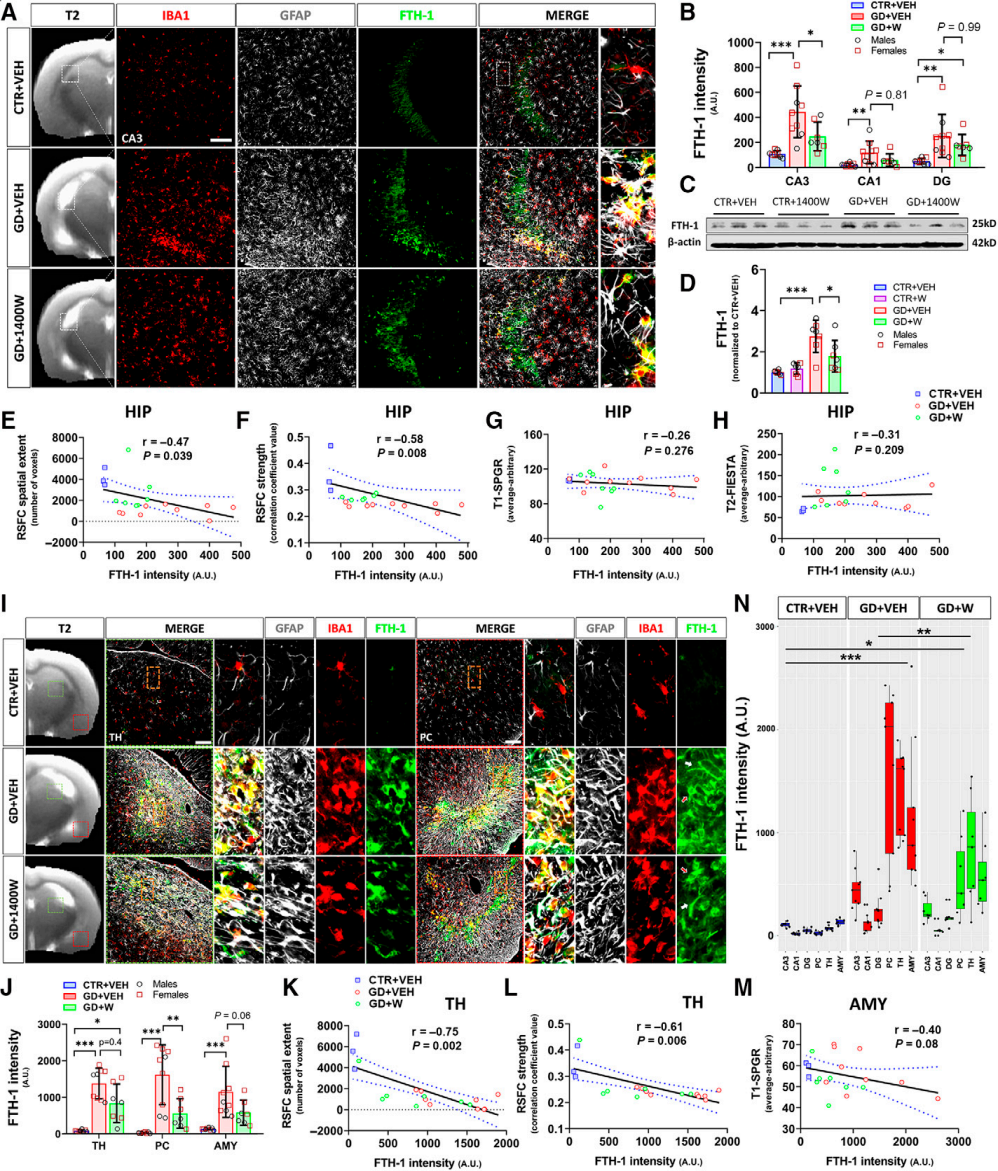
1400W suppresses GD-induced chronic iron accumulation in the brain. (A) Comparison of triple immunolabeling of iron-storage protein ferritin, (FTH-1), astrocytic (GFAP), and microglial (IBA1) markers in the hippocampus in control, GD+VEH, and GD+1400W groups. (B) FTH-1 quantification. (C) Representative hippocampal western blot of FTH-1. (D) Densitometry analysis of FTH-1 expression showing a significant reduction in GD+1400W relative to GD+VEH. (E and F) Spearman analyses showing a significant negative relationship between RSFC hippocampal (HIP) network for spatial extent (E) and strength (F) versus hippocampal FTH-1 intensity. (G and H) Spearman analyses demonstrating no significant relationship between hippocampal FTH-1 intensity and T1-SPGR (G) or T2-FIESTA intensity (H). (I) Representative images of FTH-1, astrocytes, and microglia in extrahippocampal regions showing the dorsal lateral thalamus (TH) and the piriform cortex (PC), comparing across all groups. In the last panel (FTH-1), white arrows indicate the colocalization of FTH-1 with GFAP in astrocytes; yellow arrows show a colocalization of FTH-1 with IBA1 in microglia. (J) Quantification of FTH-1 fluorescence intensity in TH, PC, and amygdala (AMY). (K and L) Spearman analyses showing a significant negative relationship between RSFC TH network for spatial extent (K) and strength (L) versus FTH-1 intensity. (M) A Spearman correlation showing a nonsignificant (*P* = 0.08) negative relationship between AMY T1-SPGR intensity and FTH-1 levels in the amygdala; *n* = 3 (CTR+VEH), 9 (GD+VEH), 7 (GD+W). (N) Linear mixed-effects analysis comparing FTH-1 intensity in all groups across all brain regions. Scale bar, 100 μm. Boxplots showed all data points and are presented as median, minimum, maximum, and all quartiles. The blue dotted lines in the correlational plots represent a 95% confidence interval for two means. *n* = 8 (CTR+VEH), 9 (GD+VEH), 7 (GD+1400W); ∗*P* < 0.05; ∗∗*P* < 0.01; ∗∗∗*P* < 0.001. Bar graphs display all data points and are expressed as mean ± S.D. No sex differences observed in all graphs. A.U., arbitrary units; CTR, control; FIESTA, fast imaging employing steady-state acquisition; GFAP, glial fibrillary acidic protein; IBA1, ionized calcium-binding adapter molecule 1; SPGR, spoiled gradient recalled; W, 1400W.

Iron accumulation in the brain has been linked to the progression of seizures in animal models (Chen et al., 2020) and human patients with epilepsy (Zimmer et al., 2021; Roggenhofer et al., 2022). To investigate whether dysregulation of iron metabolism occurs in GD-induced epilepsy and the effects of iNOS inhibition, we evaluated the levels of ferritin (FTH-1), an iron-storage protein, at 3.5 months post GD intoxication. GD exposure upregulated the expression of FTH-1 in the hippocampus, which was mostly localized in microglia and astrocytes (Fig. 4, A and B). Histologically, the GD+1400W group only had reduced FTH-1 intensity in the CA3 region relative to GD+VEH animals (Fig. 4B). Hippocampal immunoblotting, however, showed a significant reduction in FTH-1 levels in 1400W-treated animals relative to vehicle after GD exposure (Fig. 4, C and D). We also correlated the average FTH-1 intensity with hippocampal MRI measures and observed a significant inverse relationship between hippocampal RSFC spatial extent and strength and average of hippocampal FTH-1 intensity (CA1, CA3, DG) (Fig. 4, E and F). There was no significant relationship, however, between hippocampal FTH-1 intensity levels and T1 decreases or T2 increases (Fig. 4, G and H). Also, FTH-1 intensity is significantly correlated with cortico-thalamic RSFC, suggesting the extension of brain functional impairment and perhaps structural damage by GD-induced hippocampal iron deposits beyond the hippocampus (Supplemental Fig. 2).

We then examined extrahippocampal areas, including the thalamus, amygdala, and piriform cortex, to compare FTH-1 levels between groups. GD exposure significantly increased FTH-1 intensity in these areas compared with controls, and animals treated with 1400W had significantly lower FTH-1 levels only in the piriform cortex (Fig. 4, I and J). Notably, correlations also existed between thalamic FTH-1 levels and RSFC for thalamus and hippocampus (Fig. 4, K and L; Supplemental Fig. 2). Although FTH-1 intensity and T1 decreases for amygdala only have partial relationship (Fig. 4M), amygdala FTH-1 intensity had significantly correlated with thalamic and hippocampal RSFC (Supplemental Fig. 2). Linear mixed-effect analysis showed a significant upregulation of FTH levels globally following GD intoxication, which was mitigated by 1400W (Fig. 4N), demonstrating the pathologic interplay between iNOS and ferritin to facilitate GD-induced structural and functional brain abnormalities.

### 1400W Mitigated GD-Induced Accumulation of Iron in Glial Cells.

**Fig. 5.**
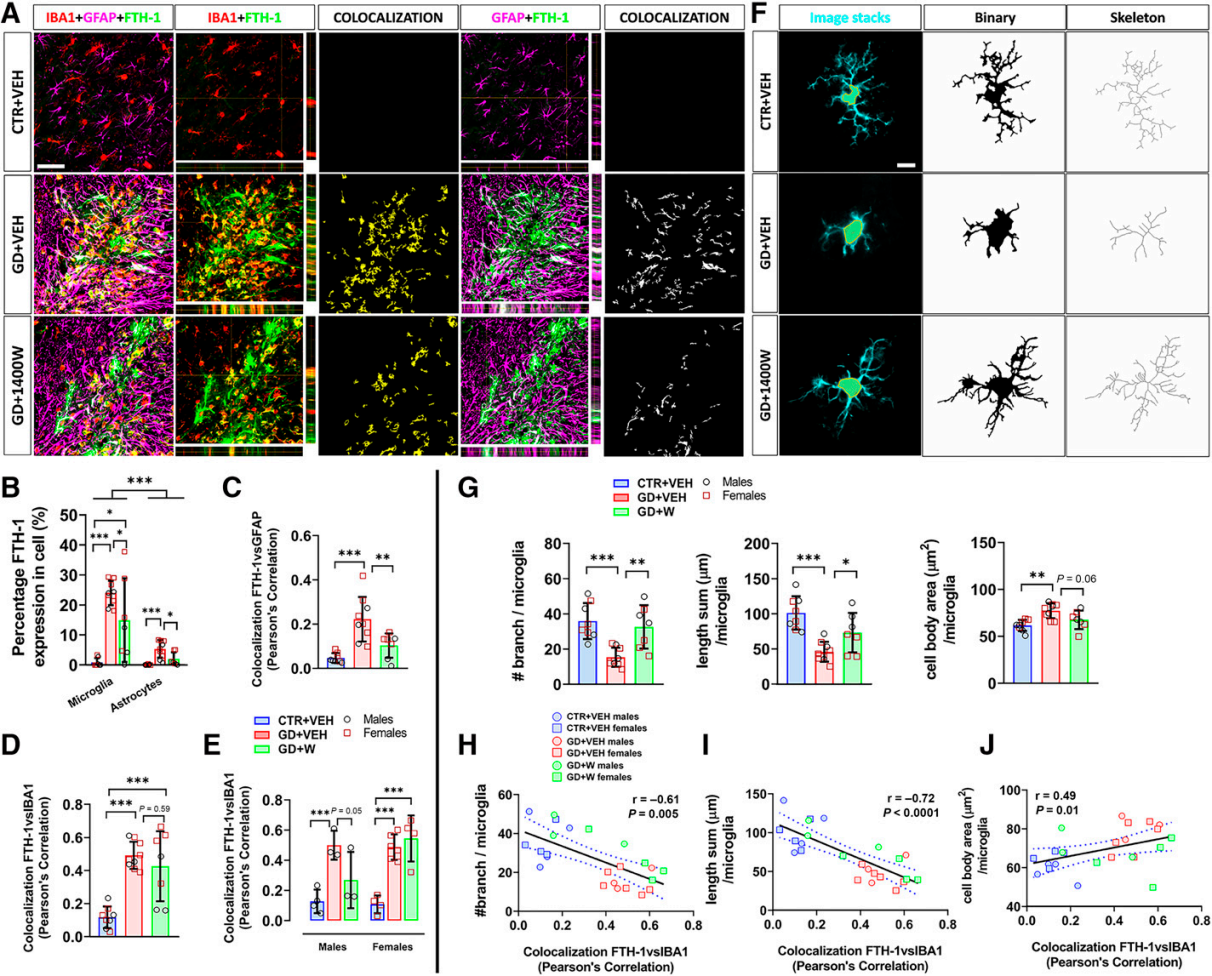
The effects of 1400W on GD-mediated pathologic iron-laden glial cells. (A) Colocalization of ferritin (FTH-1, green) and microglia (red-yellow) or astrocytes (purple-white) in the brain sections across all the groups. The merged panel includes orthogonal images of reconstructed three-dimensional views for each overlay. Colocalization analysis was carried out to determine the pixel intensity correlation between FTH-1 and microglia (yellow) as well as FTH-1 and astrocytes (white). Scale bar, 100 μm. (B) Percentage of area for the levels of FTH-1 colocalizing with microglia or astrocytes. (C and D) Colocalization quantification by Pearson’s correlation coefficient for FTH-1 versus astrocytes (C) and FTH-1 versus microglia (D). Sex interaction was observed in (D); therefore, the data for each sex is shown in (E). (F) Representative images showing the microglial morphology (IBA1, cyan) with the area of the cell body highlighted (shaded orange). Scale bar, 5 μm. (G) Morphometric analysis of microglia was performed from transformed binary (black) and skeleton (gray) for cell body area (F), number of branches, and branch length sum comparing across all groups. (H–J) Spearman correlation analysis between microglial FTH-1 levels and number of microglial branches (H), the length of branches (I) showing significantly negative relationships, and a nonsignificant relationship with the cell body area (J). The blue dotted lines in the correlational plots represent a 95% confidence interval for two means. *n* = 8 (CTR+VEH), 9 (GD+VEH), 7 (GD+1400W); ∗*P* < 0.05; ∗∗*P* < 0.01; ∗∗∗*P* < 0.001. Bar graphs display all data points and are expressed as mean ± S.D. No sex differences observed in all graphs except for (D). CTR, control; IBA1, ionized calcium-binding adapter molecule 1.

Iron-induced ferroptosis is driven by reactive microglia and astrocytes in neurodegenerative diseases, including epilepsy (Zimmer et al., 2021; Ryan et al., 2023). We evaluated the extent of glial cell–induced iron deposition by performing a colocalization study measuring the degree of overlaps between FTH-1 fluorescence with microglial and astrocytic markers (Fig. 5A). The FTH-1 predominantly colocalized with microglia relative to astrocytes after GD intoxication (Fig. 5B). GD+1400W animals had a significantly lower percentage of FTH-1 in both microglia and astrocytes than GD+VEH animals (Fig. 5B). Pearson’s colocalization analysis revealed that 1400W reduced astrocytic FTH-1 but did not significantly rescue FTH-1 buildup in microglia (Fig. 5, C and D). Interestingly, there was a significant interaction between sex and treatment of 1400W, with 1400W reducing ferritin levels in males but not in females (Fig. 5E). Considering the predominant role of microglial FTH-1 in pathogenesis, we assessed the relationships between FTH-1 levels and morphologic alterations in microglia. First, we found that GD exposure resulted in significantly less ramified microglia with enlarged soma compared with control animals, and 1400W mitigated these GD-induced morphologic changes (Fig. 5, F and G). Second, FTH-1 levels in microglia significantly correlated with the alteration in their morphology, indicating a major role of iron overload in altering microglial phenotypes (Fig. 5, H–J).

### The Impact of 1400W on GD-Induced Nitroxidative Stress and Serum Ferritin Levels.

**Fig. 6.**
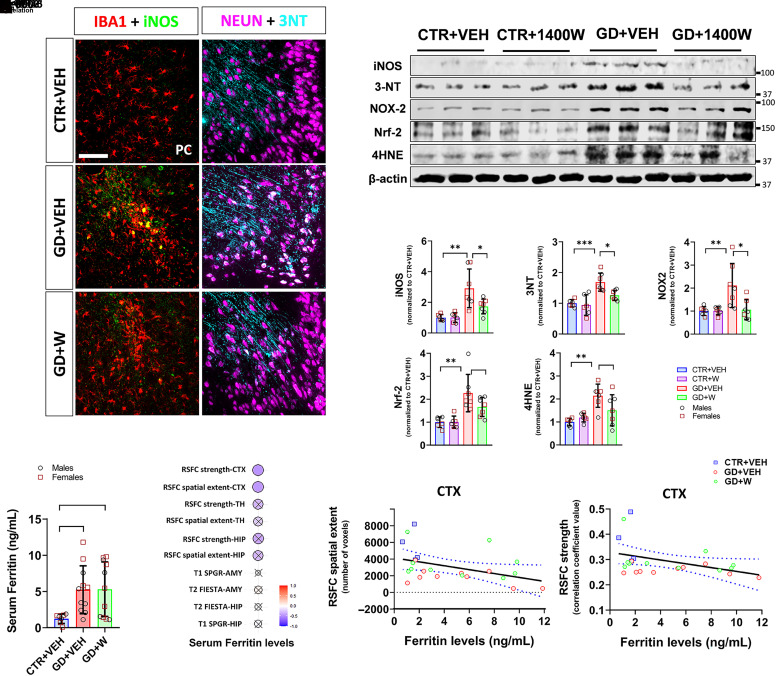
The effects of 1400W on GD-induced RNS/ROS, lipid peroxidation, and increased serum ferritin levels. (A) The panels in the left column are the representative images of double immunolabeling for iNOS (green) and microglial marker IBA1 (red). Right column images represent double immunolabeling for 3NT (bluish green) and neuronal marker NeuN (pink). Scale bar, 100 μm. (B) Representative hippocampal (HIP) immunoblots for RNS/ROS and lipid peroxidation markers. (C–G) Densitometric analysis of iNOS (C), 3NT (D), GP91^phox^ (NOX2) (E), Nrf-2 (F), and 4HNE (G) expression in the hippocampus; *n* = 6 (CTR+VEH), 6 (CTR+W), 7 (GD+VEH), 7 (GD+W). (H) Serum ferritin levels by ELISA showing an increase of ferritin levels at 3.5 months post GD exposure; *n* = 7 (CTR+VEH), 11 (GD+VEH), 11 (GD+W). (I) Spearman correlation matrix showing the relationship between serum ferritin levels with the structural, functional MRI parameters. Black crosses (X) in circles represent nonsignificant correlations; *n* = 4 (CTR+VEH), 9 (GD+VEH), 10 (GD+W). (J and K) Spearman correlation analyses showing a significant inverse relationship between serum ferritin levels and cortical (CTX) RSFC spatial extent (J) or strength (K). The blue dotted lines in the correlational plots represent a 95% confidence interval for two means. ∗*P* < 0.05; ∗∗*P* < 0.01; ∗∗∗*P* < 0.001. Bar graphs display all data points and are expressed as mean ± S.D. No sex differences observed in all graphs. AMY, amygdala; CTR, control; IBA1, ionized calcium-binding adapter molecule 1; W, 1400W.

Excessive ROS/RNS generation has been linked to epilepsy and seizure-related disorders (Ryan et al., 2014; Puttachary et al., 2016b), including OPNA-induced neurotoxicity (Pazdernik et al., 2001; Pearson and Patel, 2016; Liang et al., 2019; Putra et al., 2020a,c). Thus, we examined nitroxidative markers and observed significant increases in levels of iNOS and protein nitration, peroxynitrite (3-nitrotyrosine; 3NT), in the hippocampus and extrahippocampal regions following GD intoxication (Fig. 6, A and B). iNOS was primarily expressed in microglia, whereas 3NT was found mainly in neurons (Fig. 6A). 1400W significantly reduced GD-induced upregulated nitroxidative markers, including iNOS, 3NT, and NOX2 (gp91^phox^ subunit), in the hippocampus. Furthermore, GD exposure also significantly elevated lipid peroxidation marker 4-hydroxynonenal (4HNE) and antioxidant marker nuclear factor erythroid 2–related factor 2 (Nrf-2). Although the GD+1400W group had relatively lower expression of hippocampal 4HNE and Nrf-2 levels than the control group, they were not statistically significant (Fig. 6, B–G). Our results suggest a possible role of nitroxidative stress in GD-induced neurotoxicity, perhaps via dysregulated iron metabolism and a protective role of 1400W.

Given the extent of elevated ferritin levels in the brain following GD exposure, we speculated that the excessive iron overload could be released into extracellular space and the bloodstream. Indeed, serum ELISA showed a significant increase in ferritin levels, consistent with the findings in the brain. However, 1400W had no effect on elevated ferritin levels in the serum post GD exposure (Fig. 6H). Correlation analysis showed significantly inverse relationships between serum ferritin levels with RSFC for the cortex, suggesting a potential use of ferritin as a peripheral biomarker for GD intoxication (Fig. 6, I–K).

### Sex Differences.

No sex differences were observed in any parameters measured in this study except for initial SE severity, which was higher in females than males (Fig. 1C), and microglial ferritin levels (Fig. 5, D and E).

## Discussion

We demonstrate robust neuroimaging to capture brain pathology in the GD-induced epilepsy model and the neuroprotective effects of an iNOS inhibitor, 1400W. Importantly, the structural and functional MRI alterations in specific brain regions are markedly associated with memory dysfunction post-GD. Prior studies have demonstrated GD-induced brain pathology in the hippocampus, thalamus, piriform cortex, and amygdala (Pazdernik et al., 2001; Aroniadou-Anderjaska et al., 2009; Marrero-Rosado et al., 2020; Reddy et al., 2020). Notably, we discovered iron buildup in these regions, which correlated with disrupted functional connectivity post-GD. Elevated nitroxidative markers were evident in GD exposure, and 1400W protected against GD-associated pathologies and functional deficits, underscoring the critical role of RNS in mediating brain damage in GD exposure, possibly through dysregulation of iron homeostasis. In addition, our study provides insights into the sensitivity of fMRI in predicting memory deficits and 1400W as an adjunct therapy for GD-induced epilepsy.

T1- or T2-weighted MRI intensities have been used to assess brain pathology in the GD model (Bhagat et al., 2001, 2005; Gullapalli et al., 2010; Reddy et al., 2020; Gage et al., 2023). Although no difference in T1 and T2 intensity was observed at the ROI levels between groups in this study, the voxel-wise analysis revealed significant T1 decreases and T2 increases after GD exposure, which was consistent with previous reports (Gullapalli et al., 2010). Notably, the protective effects of 1400W on T1-T2 changes were also evident with this analysis, suggesting that voxel level analysis increased MRI sensitivity (Ashburner and Friston, 2001). The failure of conventional ROI analysis is partly owing to the overt soman effects that are selective to groups of voxels with a given ROI. Collectively, the iNOS-targeted intervention appeared to significantly rescue GD-induced structural brain atrophy and neurodegeneration, shown by the enhancement of T1 and T2 MRI changes.

fMRI studies in epilepsy demonstrate seizure-associated altered functional neuronal circuitry across multiple brain regions (Barron et al., 2014; Gill et al., 2016; Pressl et al., 2019). Owing to its capacity to reveal subtle changes in brain circuits (Woodward and Cascio, 2015), resting-state fMRI was used to assess the extent of neuronal connectivity in GD toxicity. We reported severe impairment of cortical and thalamic networks post-GD. 1400W rescued the cortical but not the thalamic network connectivity, suggesting that an alternative target may be involved in regulating the thalamic network (Jo et al., 2019). In addition, we found no correlation between motor learning performance and MRI measures associated with this task, suggesting the involvement of more complex and broader brain regions beyond the cortical network (Scholz et al., 2015). Interestingly, the degree of thalamic connectivity is reflected in the thalamic T1 and T2 intensity, indicating the overlap between fMRI and structural MRI parameters. Overall, we show interdependencies between MRI modalities in the thalamocortical network and associated structures after GD exposure and the effects of 1400W.

Structural and functional changes in the hippocampus have been extensively reported in epilepsy (Houser, 1992; Scharfman et al., 2002). Hippocampal disorganization is reportedly associated with seizure severity and cognitive impairment after GD intoxication (Philippens et al., 1992; Marrero-Rosado et al., 2020; Reddy et al., 2020). We demonstrate that GD severely disrupts hippocampal connectivity and associated memory function on NOR, whereas 1400W mitigates these deficits (Fig. 3). The neuroprotection and reversal of GD-induced cognitive dysfunction through restoration of hippocampal connectivity by 1400W may have reduced the spontaneous seizures, or vice versa, as demonstrated in our previous study (Vasanthi et al., 2023). Indeed, functional connectivity of the hippocampus strongly correlated with memory performances in NOR and hippocampal T1 and T2 intensities. However, T1 and T2 intensities for the hippocampus had no relationships with the discrimination index in NOR, probably due to minimal changes in T1 and T2 values, implying the limitation of this approach. This finding does not entirely contradict previous reports showing a positive correlation between memory performance and T1/T2 features since the time points for MRI acquisition post-GD were different (Reddy et al., 2020). Overall, our findings suggest that hippocampal RSFC may be a useful indicator of hippocampal pathology and memory impairment after GD toxicity and intervention by 1400W.

The extra hippocampal region, especially the amygdala, is also linked to fear learning and memory deficits in epilepsy (Phillips and LeDoux, 1992), including organophosphate-induced epilepsy (Philippens et al., 1992; Flannery et al., 2016; Guignet et al., 2020). We found that GD toxicity significantly impaired fear memory but not learning, whereas 1400W had no significant impact. The complex interaction between the hippocampus and amygdala in fear memory (Phillips and LeDoux, 1992) may contribute to the inadequate effects of 1400W in rescuing GD-induced fear memory deficits. 1400W effect may likely be limited to the cellular level rather than at synapses where neuronal nitric oxide synthase and Src kinases could play a more significant role in fear memory (Isosaka et al., 2009; Cai et al., 2018). We also found that fear memory performances correlate strongly with functional connectivity and microstructural changes in the hippocampus. T2 intensity for the amygdala is also significantly correlated with fear memory. Surprisingly, 1400W improved fear memory when simultaneously accounting for the changes in amygdala T2 intensities. We also discovered that the relationships between memory functions and MRI measures exist beyond previously reported brain regions. For instance, fear memory functions also have a strong positive correlation with cortical and thalamic connectivity, whereas NOR-related memory tasks have a strong connection with the cortical, but not thalamic, network (Fig. 4K), indicating robust, yet complex, interconnections between individual brain networks to facilitate intricate memory functions and retrieval.

Iron overload–mediated neurodegeneration, ferroptosis, is linked to pathologic MRI findings (Zhang et al., 2014; Roggenhofer et al., 2022). Seizures aggravate iron accumulation in the brain in animal models (Chen et al., 2020), the GD model (Pazdernik et al., 2001), and human epilepsy (Zimmer et al., 2021), resulting in cognitive impairment. Our study showed that GD intoxication increased ferritin levels in the serum and brain, particularly in regions with prominent pathology on MRI and associated behavioral dysfunction. This finding suggests a possible pathomechanism by which excessive iron buildup may mediate neuronal damage, leading to cognitive deficits in GD exposure. Ferritin was primarily localized within microglia and astrocytes, consistent with previous findings (Zimmer et al., 2021; Ryan et al., 2023). 1400W protected against GD-induced ferritin accumulation in the hippocampus and piriform cortex, indicating that blocking iNOS can mitigate iron-mediated neurotoxicity in GD intoxication. Additionally, the ferritin levels are strongly reflective of functional connectivity in the brain fMRI, but not of T1 and T2 intensity, demonstrating the sensitivity of resting-state fMRI in predicting the extent of brain pathology post-GD. Interestingly, ferritin levels in the piriform cortex were highly reflected in the functional connectivity of the thalamus, cortex, and hippocampus, indicating widespread network dysfunction in GD intoxication. Moreover, reduced connectivity in these regions approximated ferritin levels in the amygdala despite no correlation with structural MRI. This study highlights the importance of using multiparametric MRI measurements to detect subtle brain changes related to disease progression in GD-induced epilepsy.

Ferritin overexpression in glial cells is a well known response to brain insults (Zimmer et al., 2021), with microglia showing higher expression than astrocytes in the chronic stage of GD-induced epilepsy. The altered morphologies strongly correlated with microglial ferritin expression, indicating the role of iron dysregulation in facilitating pathogenic phenotypes (Zimmer et al., 2021; Ryan et al., 2023). 1400W reduced ferritin upregulation in both microglia and astrocytes, with significant protective effects observed in astrocytes, implying the differential effects of nitroxidative stress on iron metabolisms in cell types. In microglia, neuroprotection of 1400W is more pronounced in males than females, suggesting that microglia in females may have a different role in iron sequestration attributed to its reactive state (O’Neill et al., 2022). In addition, this could be due to the more severe SE experienced by females than in males in response to acute exposure to GD (Fig. 1C). However, our previous study reported no differences in EEG-based SE severity between sexes following GD intoxication. This may suggest that some of the exaggerated behavioral seizures in females could be due to the peripheral effects of OPNA rather than the central effects (Gage et al., 2022; Rao et al., 2022). Therefore, behavioral SE severity in females in response to an OPNA should be carefully interpreted to delineate differential pathology observed in both sexes if one exists.

Dysregulated iron metabolism can lead to neurodegeneration and neuroinflammation through nitroxidative pathways (Zimmer et al., 2020, 2021; Ryan et al., 2023). Previous studies have shown the role of these pathways in the pathogenesis of epilepsy in nerve agent models (Pazdernik et al., 2001; Pearson and Patel, 2016; Liang et al., 2019; Putra et al., 2020a,c). Chronic exposure to GD increased iNOS and 3NT levels in the hippocampus, indicating high degrees of protein nitration related to neurodegeneration and neuroinflammation. 1400W suppressed these GD-induced RNS, consistent with our earlier rat OPNA model of epilepsy (Putra et al., 2020c). 1400W also mitigated the increased NOX2-mediated oxidative stress and, partly, lipid peroxidation (4HNE) after GD intoxication, highlighting iNOS also as a regulator of ROS. Partial rescue of lipid peroxidation by 1400W suggests the role of other biologic factors (Ayala et al., 2014). These observations suggest that soman-induced seizures facilitate the upregulation of iNOS, increasing OONO^-^, which then leads to further ROS production. Consequently, a substantial ROS/RNS accumulation eventually promotes ferroptosis, thus resulting in neuroinflammation and neurodegeneration (Chen et al., 2020; Tang et al., 2021). Therefore, iNOS-targeted therapy mitigates GD-induced long-term neurotoxicity, potentially through iron-regulated nitroxidative pathways. Moreover, we found upregulated ferritin levels in serum during the chronic stage of epilepsy following GD exposure, whereas 1400W had no effect on serum ferritin levels at the time point tested, which was about 3 months post-treatment. Interestingly, the levels of ferritin had an inverse correlation with functional cortical connectivity, suggesting ferritin as a diagnostic and prognostic biomarker in epilepsy.

One limitation of this study is the absence of an MRI assessment for the effects of 1400W in healthy rats. However, considering that iNOS is only upregulated under pathologic conditions, an additional healthy control group with 1400W was deemed redundant for MRI studies. Moreover, 1400W treatment in control animals did not change the basal ferritin levels, nitroxidative markers (Figs. 4 and 6), or behavioral or cytokine profiles compared to naïve controls (Vasanthi et al., 2023). Although soman-induced neuronal death and rescue by 1400W was evident in our previous study (Vasanthi et al., 2023), the current study did not examine the relationship between neurodegeneration and MRI parameters, which might exist considering the positive correlation between ferroptosis and MRI findings. Nevertheless, it would be interesting to further assess these interrelations involving ferroptosis, neurodegeneration, and MRI outcomes. In this study, we also could not correlate MRI outcomes with the frequency of spontaneous seizures, which will be considered in future studies by implanting telemetry devices in the same animals after completing MRI acquisition. Furthermore, this study concluded that sex does not modify the treatment effects of 1400W or GD in all analyses except for microglial ferritin, which may be attributed to sex differences in microglial iron metabolism. GD exposure induced brain damage in both sexes despite differences in the initial seizure severities, and 1400W protected against GD-induced pathology in both sexes. It is also important to note that isoflurane can affect the amplitude of BOLD fluctuation in fMRI despite having no effects on T1 and T2 contrast (Wu et al., 2016). However, the spatial topology of RSFC remains the same across awake or anesthetized states (Hori et al., 2020). Thus, alterations in RSFC topology most likely reflect GD-induced changes in brain function, albeit with relatively reduced effect sizes than without anesthesia.

In conclusion, our study demonstrates the versatility and sensitivity of structural and fMRI to identify unique brain abnormalities in the chronic stage of epilepsy in the rat GD model and the protective effects of 1400W. These MRI measures correlated with behavioral and molecular markers related to GD-induced epilepsy. Our study also demonstrated the role of iron dysregulation and nitroxidative stress in GD-induced brain functional deficits, suggesting a potential mechanism by which iNOS inhibition may mediate brain protection. Taken together, iNOS inhibition after acute exposure to organophosphate nerve agent protects the brain by targeting ferroptosis, and both structural and functional MRI serve as reliable neuroimaging tools to study the microstructural and functional changes that reflect the memory outcomes and brain pathology in GD intoxication.

## Abbreviations

3NT: 3-nitrotyrosine
4HNE: 4-hydroxynonenal
BOLD: blood oxygenation level–dependent
DI: discrimination index
EEG: electroencephalogram
fMRI: functional magnetic resonance imaging
FT: freezing time
FTH-1: ferritin heavy chain
GD: soman
IACUC: Institutional Animal Care and Use Committee
iNOS: inducible nitric oxide synthase
MRI: magnetic resonance imaging
NIH: National Institutes of Health
NOR: novel object recognition
OPNA: organophosphate nerve agent
RNS: reactive nitrogen species
ROI: region of interest
ROS: reactive oxygen species
RSFC: resting-state functional connectivity
SE: status epilepticus
VEH: vehicle

## References

[B1] Aroniadou-Anderjaska V Figueiredo TH Apland JP Qashu F Braga MFM (2009) Primary brain targets of nerve agents: the role of the amygdala in comparison to the hippocampus. Neurotoxicology 30:772–776. 19591865 10.1016/j.neuro.2009.06.011 PMC2761531

[B2] Ashburner J Friston KJ (2001) Why voxel-based morphometry should be used. Neuroimage 14:1238–1243. 11707080 10.1006/nimg.2001.0961

[B3] Ayala A Muñoz MF Argüelles S (2014) Lipid peroxidation: production, metabolism, and signaling mechanisms of malondialdehyde and 4-hydroxy-2-nonenal. Oxid Med Cell Longev 2014:360438. 24999379 10.1155/2014/360438 PMC4066722

[B4] Barron DS Tandon N Lancaster JL Fox PT (2014) Thalamic structural connectivity in medial temporal lobe epilepsy. Epilepsia 55:e50–e55. 24802969 10.1111/epi.12637 PMC4791041

[B5] Beamer E Otahal J Sills GJ Thippeswamy T (2012) N (w) -propyl-L-arginine (L-NPA) reduces status epilepticus and early epileptogenic events in a mouse model of epilepsy: behavioural, EEG and immunohistochemical analyses. Eur J Neurosci 36:3194–3203. 22943535 10.1111/j.1460-9568.2012.08234.x

[B6] Bhagat YA Obenaus A Hamilton MG Kendall EJ (2001) Magnetic resonance imaging predicts neuropathology from soman-mediated seizures in the rodent. Neuroreport 12:1481–1487. 11388434 10.1097/00001756-200105250-00037

[B7] Bhagat YA Obenaus A Hamilton MG Mikler J Kendall EJ (2005) Neuroprotection from soman-induced seizures in the rodent: evaluation with diffusion- and T2-weighted magnetic resonance imaging. Neurotoxicology 26:1001–1013. 15982742 10.1016/j.neuro.2005.04.006

[B8] Boux F Forbes F Collomb N Zub E Mazière L de Bock F Blaquiere M Stupar V Depaulis A Marchi N, (2021) Neurovascular multiparametric MRI defines epileptogenic and seizure propagation regions in experimental mesiotemporal lobe epilepsy. Epilepsia 62:1244–1255. 33818790 10.1111/epi.16886

[B9] Cai C-Y Chen C Zhou Y Han Z Qin C Cao B Tao Y Bian X-L Lin Y-H Chang L, (2018) PSD-95-nNOS Coupling Regulates Contextual Fear Extinction in the Dorsal CA3. Sci Rep 8:12775. 30143658 10.1038/s41598-018-30899-4 PMC6109109

[B10] Chen S Chen Y Zhang Y Kuang X Liu Y Guo M Ma L Zhang D Li Q (2020) Iron Metabolism and Ferroptosis in Epilepsy. Front Neurosci 14:601193. 33424539 10.3389/fnins.2020.601193 PMC7793792

[B11] Collombet J-M (2011) Nerve agent intoxication: recent neuropathophysiological findings and subsequent impact on medical management prospects. Toxicol Appl Pharmacol 255:229–241. 21791221 10.1016/j.taap.2011.07.003

[B12] Cox RW (1996) AFNI: software for analysis and visualization of functional magnetic resonance neuroimages. Comput Biomed Res 29:162–173. 8812068 10.1006/cbmr.1996.0014

[B13] Duncan JS Papademetris X Yang J Jackowski M Zeng X Staib LH (2004) Geometric strategies for neuroanatomic analysis from MRI. Neuroimage 23(Suppl 1, Suppl 1)S34–S45. 15501099 10.1016/j.neuroimage.2004.07.027 PMC2832750

[B14] Dunn KW Kamocka MM McDonald JH (2011) A practical guide to evaluating colocalization in biological microscopy. Am J Physiol Cell Physiol 300:C723–C742. 21209361 10.1152/ajpcell.00462.2010 PMC3074624

[B15] Flannery BM Bruun DA Rowland DJ Banks CN Austin AT Kukis DL Li Y Ford BD Tancredi DJ Silverman JL, (2016) Persistent neuroinflammation and cognitive impairment in a rat model of acute diisopropylfluorophosphate intoxication. J Neuroinflammation 13:267. 27733171 10.1186/s12974-016-0744-y PMC5062885

[B16] Fu C Aisikaer A Chen Z Yu Q Yin J Yang W (2021) Different Functional Network Connectivity Patterns in Epilepsy: A Rest-State fMRI Study on Mesial Temporal Lobe Epilepsy and Benign Epilepsy With Centrotemporal Spike. Front Neurol 12:668856. 34122313 10.3389/fneur.2021.668856 PMC8193721

[B17] Gage M Rao NS Samidurai M Putra M Vasanthi SS Meyer C Wang C Thippeswamy T (2022) Soman (GD) Rat Model to Mimic Civilian Exposure to Nerve Agent: Mortality, Video-EEG Based *Status Epilepticus* Severity, Sex Differences, Spontaneously Recurring Seizures, and Brain Pathology. Front Cell Neurosci 15:798247. 35197823 10.3389/fncel.2021.798247 PMC8859837

[B18] Gage M Vasanthi SS Meyer CM Rao NS Thedens DR Kannurpatti SS Thippeswamy T (2023) Sex based structural and functional MRI outcomes in the rat brain after soman (GD) exposure induced status epilepticus. Epilepsia Open 8:399–410. 36718979 10.1002/epi4.12701 PMC10235578

[B19] Gill RS Mirsattari SM Leung LS (2016) Resting state functional network disruptions in a kainic acid model of temporal lobe epilepsy. Neuroimage Clin 13:70–81. 27942449 10.1016/j.nicl.2016.11.002 PMC5133653

[B20] Guignet M Dhakal K Flannery BM Hobson BA Zolkowska D Dhir A Bruun DA Li S Wahab A Harvey DJ, (2020) Persistent behavior deficits, neuroinflammation, and oxidative stress in a rat model of acute organophosphate intoxication. Neurobiol Dis 133:104431. 30905768 10.1016/j.nbd.2019.03.019 PMC6754818

[B21] Gullapalli RP Aracava Y Zhuo J Helal Neto E Wang J Makris G Merchenthaler I Pereira EFR Albuquerque EX (2010) Magnetic resonance imaging reveals that galantamine prevents structural brain damage induced by an acute exposure of guinea pigs to soman. Neurotoxicology 31:67–76. 19782102 10.1016/j.neuro.2009.09.004

[B22] Henretig FM Kirk MA McKay Jr CA (2019) Hazardous Chemical Emergencies and Poisonings. N Engl J Med 380:1638–1655. 31018070 10.1056/NEJMra1504690

[B23] Hobson BA Rowland DJ Supasai S Harvey DJ Lein PJ Garbow JR (2018) A magnetic resonance imaging study of early brain injury in a rat model of acute DFP intoxication. Neurotoxicology 66:170–178. 29183789 10.1016/j.neuro.2017.11.009 PMC5940565

[B24] Hori Y Schaeffer DJ Gilbert KM Hayrynen LK Cléry JC Gati JS Menon RS Everling S (2020) Altered Resting-State Functional Connectivity Between Awake and Isoflurane Anesthetized Marmosets. Cereb Cortex 30:5943–5959. 32556184 10.1093/cercor/bhaa168 PMC7899065

[B25] Houser CR (1992) Morphological changes in the dentate gyrus in human temporal lobe epilepsy. Epilepsy Res Suppl 7:223–234. 1466768

[B26] Isosaka T Kida S Kohno T Hattori K Yuasa S (2009) Hippocampal Fyn activity regulates extinction of contextual fear. Neuroreport 20:1461–1465. 19752763 10.1097/WNR.0b013e32833203a8

[B27] Jett DA (2016) The NIH Countermeasures Against Chemical Threats Program: overview and special challenges. Ann N Y Acad Sci 1374:5–9. 27398820 10.1111/nyas.13179 PMC4943675

[B28] Jett DA Spriggs SM (2020) Translational research on chemical nerve agents. Neurobiol Dis 133:104335. 30468862 10.1016/j.nbd.2018.11.020

[B29] Jo HJ Kenny-Jung DL Balzekas I Benarroch EE Jones DT Brinkmann BH Matt Stead S Van Gompel JJ Welker KM Worrell GA (2019) Nuclei-specific thalamic connectivity predicts seizure frequency in drug-resistant medial temporal lobe epilepsy. Neuroimage Clin 21:101671. 30642762 10.1016/j.nicl.2019.101671 PMC6412104

[B30] Kannurpatti SS Sanganahalli BG Herman P Hyder F (2015) Role of mitochondrial calcium uptake homeostasis in resting state fMRI brain networks. NMR Biomed 28:1579–1588. 26439799 10.1002/nbm.3421 PMC4621005

[B31] Kilkenny C Browne WJ Cuthill IC Emerson M Altman DG (2010) Improving bioscience research reporting: the ARRIVE guidelines for reporting animal research. PLoS Biol 8:e1000412. 20613859 10.1371/journal.pbio.1000412 PMC2893951

[B32] Kuzniecky RI Bilir E Gilliam F Faught E Palmer C Morawetz R Jackson G (1997) Multimodality MRI in mesial temporal sclerosis: relative sensitivity and specificity. Neurology 49:774–778. 9305339 10.1212/wnl.49.3.774

[B33] Liang L-P Pearson-Smith JN Huang J Day BJ Patel M (2019) Neuroprotective effects of a catalytic antioxidant in a rat nerve agent model. Redox Biol 20:275–284. 30384261 10.1016/j.redox.2018.10.010 PMC6215030

[B34] Marrero-Rosado BM de Araujo Furtado M Kundrick ER Walker KA Stone MF Schultz CR Nguyen DA Lumley LA (2020) Ketamine as adjunct to midazolam treatment following soman-induced status epilepticus reduces seizure severity, epileptogenesis, and brain pathology in plasma carboxylesterase knockout mice. Epilepsy Behav 111:107229. 32575012 10.1016/j.yebeh.2020.107229 PMC7541728

[B35] McDonough Jr JH Shih TM (1997) Neuropharmacological mechanisms of nerve agent-induced seizure and neuropathology. Neurosci Biobehav Rev 21:559–579. 9353792 10.1016/s0149-7634(96)00050-4

[B36] Miyaki K Nishiwaki Y Maekawa K Ogawa Y Asukai N Yoshimura K Etoh N Matsumoto Y Kikuchi Y Kumagai N, (2005) Effects of sarin on the nervous system of subway workers seven years after the Tokyo subway sarin attack. J Occup Health 47:299–304. 16096354 10.1539/joh.47.299

[B37] O’Brien CB Baghdoyan HA, and Lydic R (2019) Computer-based Multitaper Spectrogram Program for Electroencephalographic Data. J Vis Exp 153:e60333 DOI: 10.3791/60333. 31789318

[B38] O’Neill E Mela V Gaban AS Bechet S McGrath A Walsh A McIntosh A Lynch MA (2022) Sex-Related Microglial Perturbation Is Related to Mitochondrial Changes in a Model of Alzheimer’s Disease. Front Cell Neurosci 16:939830. 35875349 10.3389/fncel.2022.939830 PMC9297004

[B40] Pazdernik TL Emerson MR Cross R Nelson SR Samson FE (2001) Soman-induced seizures: limbic activity, oxidative stress and neuroprotective proteins. J Appl Toxicol 21 (Suppl 1):S87–S94. 11920927 10.1002/jat.818

[B41] Pearson JN Patel M (2016) The role of oxidative stress in organophosphate and nerve agent toxicity. Ann N Y Acad Sci 1378:17–24. 27371936 10.1111/nyas.13115 PMC5063722

[B42] Philippens IHCHM Melchers BPC de Groot DMG Wolthuis OL (1992) Behavioral performance, brain histology, and EEG sequela after immediate combined atropine/diazepam treatment of soman-intoxicated rats. Pharmacol Biochem Behav 42:711–719. 1513852 10.1016/0091-3057(92)90019-c

[B43] Phillips RG LeDoux JE (1992) Differential contribution of amygdala and hippocampus to cued and contextual fear conditioning. Behav Neurosci 106:274–285. 1590953 10.1037//0735-7044.106.2.274

[B44] Pressl C Brandner P Schaffelhofer S Blackmon K Dugan P Holmes M Thesen T Kuzniecky R Devinsky O Freiwald WA (2019) Resting state functional connectivity patterns associated with pharmacological treatment resistance in temporal lobe epilepsy. Epilepsy Res 149:37–43. 30472489 10.1016/j.eplepsyres.2018.11.002 PMC6483378

[B45] Putra M Gage M Sharma S Gardner C Gasser G Anantharam V Thippeswamy T (2020a) Diapocynin, an NADPH oxidase inhibitor, counteracts diisopropylfluorophosphate-induced long-term neurotoxicity in the rat model. Ann N Y Acad Sci 1479:75–93. 32037612 10.1111/nyas.14314 PMC7415478

[B46] Putra M Puttachary S Liu G Lee G Thippeswamy T (2020b) Fyn-tau Ablation Modifies PTZ-Induced Seizures and Post-seizure Hallmarks of Early Epileptogenesis. Front Cell Neurosci 14:592374. 33363455 10.3389/fncel.2020.592374 PMC7752812

[B47] Putra M Sharma S Gage M Gasser G Hinojo-Perez A Olson A Gregory-Flores A Puttachary S Wang C Anantharam V, (2020c) Inducible nitric oxide synthase inhibitor, 1400W, mitigates DFP-induced long-term neurotoxicity in the rat model. Neurobiol Dis 133:104443. 30940499 10.1016/j.nbd.2019.03.031 PMC6768773

[B48] Puttachary S Sharma S Thippeswamy A Thippeswamy T (2016a) Immediate epileptogenesis: Impact on brain in C57BL/6J mouse kainate model. Front Biosci (Elite Ed) 8:390–411. 27100347 10.2741/e775

[B49] Puttachary S Sharma S Verma S Yang Y Putra M Thippeswamy A Luo D Thippeswamy T (2016b) 1400W, a highly selective inducible nitric oxide synthase inhibitor is a potential disease modifier in the rat kainate model of temporal lobe epilepsy. Neurobiol Dis 93:184–200. 27208748 10.1016/j.nbd.2016.05.013

[B50] Rao NS Meyer C Vasanthi SS Massey N Samidurai M Gage M Putra M Almanza AN Wachter L Thippeswamy T (2022) DFP-Induced Status Epilepticus Severity in Mixed-Sex Cohorts of Adult Rats Housed in the Same Room: Behavioral and EEG Comparisons. Front Cell Dev Biol 10:895092. 35620057 10.3389/fcell.2022.895092 PMC9127803

[B51] Reddy SD Wu X Kuruba R Sridhar V Reddy DS (2020) Magnetic resonance imaging analysis of long-term neuropathology after exposure to the nerve agent soman: correlation with histopathology and neurological dysfunction. Ann N Y Acad Sci 1480:116–135. 32671850 10.1111/nyas.14431 PMC7708405

[B52] Roggenhofer E Toumpouli E Seeck M Wiest R Lutti A Kherif F Novy J Rossetti AO Draganski B (2022) Clinical phenotype modulates brain’s myelin and iron content in temporal lobe epilepsy. Brain Struct Funct 227:901–911. 34817680 10.1007/s00429-021-02428-z PMC8930791

[B53] Ryan K Liang L-P Rivard C Patel M (2014) Temporal and spatial increase of reactive nitrogen species in the kainate model of temporal lobe epilepsy. Neurobiol Dis 64:8–15. 24361554 10.1016/j.nbd.2013.12.006 PMC4872513

[B54] Ryan SK Zelic M Han Y Teeple E Chen L Sadeghi M Shankara S Guo L Li C Pontarelli F, (2023) Microglia ferroptosis is regulated by SEC24B and contributes to neurodegeneration. Nat Neurosci 26:12–26. 36536241 10.1038/s41593-022-01221-3 PMC9829540

[B55] Scharfman HE Sollas AL Goodman JH (2002) Spontaneous recurrent seizures after pilocarpine-induced status epilepticus activate calbindin-immunoreactive hilar cells of the rat dentate gyrus. Neuroscience 111:71–81. 11955713 10.1016/s0306-4522(01)00599-1

[B56] Schartz ND Wyatt-Johnson SK Price LR Colin SA Brewster AL (2018) Status epilepticus triggers long-lasting activation of complement C1q-C3 signaling in the hippocampus that correlates with seizure frequency in experimental epilepsy. Neurobiol Dis 109 (Pt A):163–173. 29074125 10.1016/j.nbd.2017.10.012

[B57] Scholz J Niibori Y W Frankland P P Lerch J (2015) Rotarod training in mice is associated with changes in brain structure observable with multimodal MRI. Neuroimage 107:182–189. 25497397 10.1016/j.neuroimage.2014.12.003

[B58] Tang D Chen X Kang R Kroemer G (2021) Ferroptosis: molecular mechanisms and health implications. Cell Res 31:107–125. 33268902 10.1038/s41422-020-00441-1 PMC8026611

[B59] Vale JA Marrs TC Maynard RL (2018) Novichok: a murderous nerve agent attack in the UK. Clin Toxicol (Phila) 56:1093–1097. 29757015 10.1080/15563650.2018.1469759

[B60] Vasanthi SS Rao NS Samidurai M Massey N Meyer C Gage M Kharate M Almanza A Wachter L Mafuta C, (2023) Disease-modifying effects of a glial-targeted inducible nitric oxide synthase inhibitor (1400W) in mixed-sex cohorts of a rat soman (GD) model of epilepsy. J Neuroinflammation 20:163. 37438764 10.1186/s12974-023-02847-1 PMC10337207

[B61] Vorland CJ (2021) Sex difference analyses under scrutiny. eLife 10:e74135. 34726155 10.7554/eLife.74135 PMC8562997

[B62] Woodward ND Cascio CJ (2015) Resting-state functional connectivity in psychiatric disorders. JAMA Psychiatry 72:743–744. 26061674 10.1001/jamapsychiatry.2015.0484 PMC4693599

[B63] Wu T-L Mishra A Wang F Yang P-F Gore JC Chen LM (2016) Effects of isoflurane anesthesia on resting-state fMRI signals and functional connectivity within primary somatosensory cortex of monkeys. Brain Behav 6:e00591. 28032008 10.1002/brb3.591 PMC5167001

[B64] Young K Morrison H (2018) Quantifying Microglia Morphology from Photomicrographs of Immunohistochemistry Prepared Tissue Using ImageJ. J Vis Exp 57648. 29939190 10.3791/57648 PMC6103256

[B65] Zhang Z Liao W Bernhardt B Wang Z Sun K Yang F Liu Y Lu G (2014) Brain iron redistribution in mesial temporal lobe epilepsy: a susceptibility-weighted magnetic resonance imaging study. BMC Neurosci 15:117. 25413842 10.1186/s12868-014-0117-3 PMC4243317

[B66] Zimmer TS Ciriminna G Arena A Anink JJ Korotkov A Jansen FE van Hecke W Spliet WG van Rijen PC Baayen JC, (2020) Chronic activation of anti-oxidant pathways and iron accumulation in epileptogenic malformations. Neuropathol Appl Neurobiol 46:546–563. 31869431 10.1111/nan.12596 PMC7308211

[B67] Zimmer TS David B Broekaart DWM Schidlowski M Ruffolo G Korotkov A van der Wel NN van Rijen PC Mühlebner A van Hecke W, (2021) Seizure-mediated iron accumulation and dysregulated iron metabolism after status epilepticus and in temporal lobe epilepsy. Acta Neuropathol 142:729–759. 34292399 10.1007/s00401-021-02348-6 PMC8423709

